# Supplementary material for: Saccharomyces boulardii (CNCM I-745) improves intestinal damage in sepsis by remodeling intestinal flora structure
Source: Front Cell Infect Microbiol. 2025 Jul 31;15:1602792. doi: 10.3389/fcimb.2025.1602792 (PMC12350329; doi:10.3389/fcimb.2025.1602792)
Supplement: Supplementary file 1 [file DataSheet1.zip › summary/summary/7_Differential_analysis/2_LEfSe_result/Group_DW0_vs_CW0_vs_BW0_vs_AW0_0.05_3.0/LEfSe_Group_DW0_vs_CW0_vs_BW0_vs_AW0.abund.pdf]

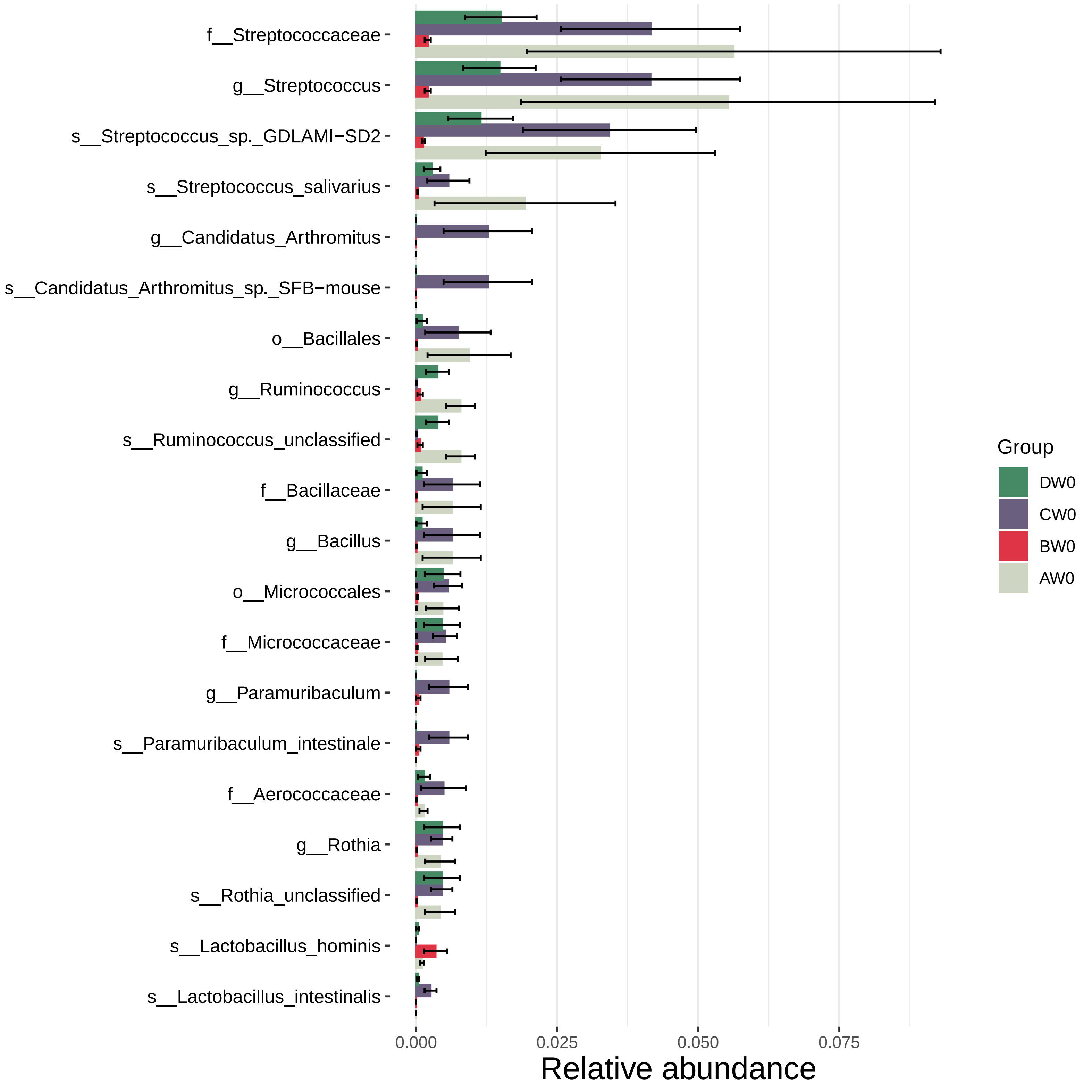

f__Streptococcaceae
g__Streptococcus
s__Streptococcus_sp._GDLAMI−SD2
s__Streptococcus_salivarius
g__Candidatus_Arthromitus
s__Candidatus_Arthromitus_sp._SFB−mouse
o__Bacillales
g__Ruminococcus
s__Ruminococcus_unclassified
f__Bacillaceae
g__Bacillus
o__Micrococcales
f__Micrococcaceae
g__Paramuribaculum
s__Paramuribaculum_intestinale
f__Aerococcaceae
g__Rothia
s__Rothia_unclassified
s__Lactobacillus_hominis
s__Lactobacillus_intestinalis
0.000
0.025
0.050
0.075
Relative abundance
Group
DW0
CW0
BW0
AW0
